# Clinimetric properties of the Chinese version of the early inflammatory arthritis detection tool

**DOI:** 10.1186/s12891-015-0706-z

**Published:** 2015-09-07

**Authors:** Ming-Chi Lu, Malcolm Koo, Ning-Sheng Lai

**Affiliations:** Division of Allergy, Immunology and Rheumatology, Dalin Tzu Chi Hospital, Buddhist Tzu Chi Medical Foundation, 2 Minsheng Road, Dalin, 62247 Taiwan; School of Medicine, Tzu Chi University, Hualien City, Taiwan; Department of Medical Research, Dalin Tzu Chi Hospital, Buddhist Tzu Chi Medical Foundation, Dalin, Taiwan; Dalla Lana School of Public Health, University of Toronto, Toronto, Ontario Canada

## Abstract

**Background:**

Timely rheumatologic referral and management are crucial for patients with potential inflammatory arthritis. To meet this need, tools such as the early inflammatory arthritis (EIA) detection tool was developed and has been evaluated in Western populations. The aims of this study were to translate the English version of the EIA detection tool to Chinese and to determine its clinimetric properties in Taiwanese patients.

**Methods:**

Twenty controls and 111 patients with established diagnosis of osteoarthritis, rheumatoid arthritis, systemic autoimmune diseases, psoriatic arthritis, and ankylosing spondylitis were recruited from a regional hospital in south Taiwan. Multivariate logistic regression analysis was used to evaluate the independent and significant variables associated with diagnosis by rheumatologists. A prediction model was also developed for differentiating between patients with inflammatory arthritis and those with non-inflammatory arthritis musculoskeletal conditions.

**Results:**

The Chinese version of the EIA detection tool showed acceptable internal consistency (KR-20 coefficient 0.78) and test-retest reliability (κ statistic ranged from 0.43 to 0.94). A prediction model consisting of three EIA detection tool items (joint pain, swelling in hands or wrists, and ever been told to have rheumatoid arthritis) and sex was able to differentiate inflammatory arthritis and non-inflammatory arthritis musculoskeletal conditions with a sensitivity of 0.84, a specificity of 0.86, a positive predictive value of 0.92, and a negative predictive value of 0.76.

**Conclusions:**

The Chinese version of the EIA detection tool showed good clinimetric properties in this study population and it can be used to differentiate Taiwanese patients with inflammatory arthritis and non-inflammatory arthritis musculoskeletal conditions.

## Background

Timely rheumatologic referral and management are crucial for patients with potential inflammatory arthritis. Early initiation of disease modifying antirheumatic drug (DMARD) has been shown to improve clinical and radiographic outcomes in patients with rheumatoid arthritis–the most common type of inflammatory arthritis [1, 2]. Ideally, treatment should be initiated within 3 to 6 months of symptom onset to reduce joint destruction [3], which would otherwise progress to functional disability with severe health and economic consequences [4, 5]. However, a population-based study in Québec, Canada showed that in 10,001 patients coded as incident rheumatoid arthritis, only 27.3 % of them consulted a rheumatologist within the next 2.5–3.5 years. Among those who consulted, it took a median of 79 days from the initial physician visit for rheumatoid arthritis to consultation with a rheumatologist [6]. Our previous study also showed that Taiwanese patients with rheumatoid arthritis had increased ambulatory visits for musculoskeletal disorders several years before their definite diagnosis of rheumatoid arthritis [7].

To be able to initiate early treatment, patients need to be identified at the earliest possible stage of their disease with appropriate tools [8]. Currently, population-based screening for preclinical phase of rheumatoid arthritis based on autoantibodies’ and cytokines’ profiles are not cost-effective due to its low prevalence. On the other hand, identification of patients mostly like to have rheumatoid arthritis at the primary care level for referral to rheumatologists is warranted because clinical outcomes can be improved with early treatment initiation [9]. One instrument for this identification purpose is the early inflammatory arthritis (EIA) detection tool, which is a 12-item patient self-administered questionnaire with yes/no questions on symptoms of arthritis, functional ability, personal and family history of rheumatoid arthritis, a diagnosis of psoriasis, and whether the symptom duration was between 6 and 52 weeks. The inflammatory arthritis dimensions and constructs of the tool were identified from a systematic review of the literature and a three-round Delphi panel consensus [10]. A scoring algorithm based on the EIA detection tool was also developed to optimize the discriminative properties for inflammation arthritis detection in rheumatology triage clinical settings. The scoring algorithm showed a sensitivity of 0.86, a specificity of 0.87, and a receiver operating characteristic plot area under the curve value of 0.92 [11]. A study of the EIA detection tool on 39 patients with early inflammatory arthritis, 43 with established inflammatory arthritis, 50 with non-inflammatory arthritis musculoskeletal conditions, and 38 with non-musculoskeletal conditions demonstrated good comprehensibility, internal consistency, and test-retest reliability. A previously developed rheumatology triage scoring algorithm, which includes age, sex, EIA detection tool items 4 (trouble making a fist), 5 (morning stiffness), 9 (ever been told to have rheumatoid arthritis), and 11 (psoriasis diagnosis) was able to differentiate early inflammatory arthritis from the other three groups, with a receiver operating characteristic plot area under the curve value of 0.83 [12]. Given the promising psychometric properties and clinical applications of the EIA detection tool in Western populations, we translated the EIA detection tool into Chinese and assessed its clinimetric properties in healthy controls and in patients with established diagnosis of different musculoskeletal disorders including osteoarthritis, rheumatoid arthritis, systemic autoimmune diseases, psoriatic arthritis, and ankylosing spondylitis.

## Methods

### Sample and data collection

The study was conducted from April 2014 to July 2014 in a regional hospital located in south Taiwan. Patients with established diagnosis of musculoskeletal disorders including osteoarthritis, rheumatoid arthritis, systemic autoimmune diseases (systemic lupus erythematosus, progressive systemic sclerosis, and Sjögren’s syndrome), psoriatic arthritis, and ankylosing spondylitis in the outpatient division of Allergy, Immunology and Rheumatology were invited to participate in the study. We used purposive recruitment to ensure that each rheumatic disease group, in addition to the control group and the osteoarthritis group, had at least 20 individuals. Participants were asked to complete a questionnaire with questions on age, sex, and a 12-item EIA detection tool in Chinese (described below). The study protocol was reviewed and approved by the institutional review board of the Dalin Tzu Chi Hospital, Buddhist Tzu Chi Medical Foundation, Taiwan (No. B10301018). All participants gave written informed consent.

### EIA detection tool

The EIA detection tool was originally developed by Bell and colleagues [10] in Canada for EIA detection in pre-primary care settings. Based on a literature review and a three-round Dephi consensus panel of EIA experts and stakeholders, an 11-question EIA detection tool suitable for self-administration was developed. The 11 questions capture seven dimensions of EIA detection, which include articular pain, swelling and stiffness, distribution of joint involvement, function, and diagnostic and family history of inflammatory arthritis. For early detection of inflammatory arthritis, the tool should be used among patients with at least 6 weeks but less than 52 weeks of musculoskeletal symptoms.

### Development of a Chinese version of EIA detection tool

Permission to translate and adapt the English version of the EIA detection tool to Chinese was obtained from Dr. Mary J. Bell of the original source. A forward and backward process was applied to translate the EIA detection tool from English into Chinese (in traditional Chinese characters). Two independent physicians who were native Chinese speakers and fluent in English produced a forward translation of the EIA detection tool with the aim of conceptual rather than literal translation. Two other bilingual physicians translated the provisional Chinese questionnaire back into English. The original English and back-translated English versions were compared for discrepancies by two authors (MCL, MK). One difference between the two versions that we decided to keep was the wording in item 3. Instead of “hands or wrists” in the original English version, we used the same wording “wrists and hands (palms and fingers)” for consistency, as in our item 2. The provisional Chinese version of the questionnaire was used for further psychometric testing.

The general comprehension of the provisional version of the questionnaire was tested on five healthy individuals with different educational levels. Based on the parts that required clarification, we had made three additional modifications. First, we changed item 7 “Are the same joints involved on both sides of your body?” to “Are the above pain or swollen conditions occur to the same joints involved on both sides of your body?”. Second, the concept of “leisure activities” was not intuitively understood by some elderly without further elaboration. The meaning of leisure activities also varies depending on the sex, age, and socioeconomic status of the respondents. Therefore, the words “leisure activities” were substituted by “other activities” in item 8. Third, we noted that a few patients without psoriasis incorrectly responded to item 11. The reason for that confusion is because the Chinese term for psoriasis contains a character that is also used for dermatophytosis. Although we included two variants of the Chinese term for psoriasis, both terms still contain one of the Chinese characters for dermatophytosis. There appears to be no simple way to correct this misnomer in the Chinese term for psoriasis.

### Statistical analysis

Comparisons of sex and age across control and rheumatic disease groups were performed using Fisher’s exact test and non-parametric Kruskal-Wallis test, respectively. Dunn-Sidak correction was applied to control for the probability of Type I errors in pairwise comparisons.

Since item 12 is a measurement of symptom duration, it was not included in the analysis of the clinimetric properties of the Chinese EIA detection tool. An overall score for the Chinese EIA detection tool for each respondent was calculated by summing the yes responses of the 11 items and then standardized the total to a final score with a range of 0 to 100. The differences across the control and the five disease groups in the Chinese EIA detection tool score were compared using the Kruskal Wallis test. Mann–Whitney test with Dunn-Sidak corrections were applied in the pairwise comparisons. The frequency distribution of each of the 11 items across the control and the five disease groups were compared using Fisher’s exact test with Dunn-Sidak correction.

Internal consistency of the Chinese EIA detection tool was examined using the Kuder-Richardson Formula 20 (KR-20), which is an equivalent of Cronbach’s alpha for binary data. Test-retest reliability of the Chinese EIA detection tool was estimated by kappa statistic, κ, which tested for agreement with regard to the presence or absence of each of the Chinese EIA detection tool item between the two time points. A mean washout period of 28 days (range 14–49 days) between the initial tool completion and the retesting was used.

A multivariate logistic regression analysis with likelihood ratio backward selection procedure was used to evaluate the independent and significant variables associated with diagnosis by rheumatologists. The independent variables evaluated in the model included age, sex, and the 11 items in the Chinese EIA detection tool.

The rheumatic disease groups were further combined into two groups: an inflammatory arthritis group (rheumatoid arthritis and psoriatic arthritis) and a non-inflammatory arthritis musculoskeletal disorders group (osteoarthritis, progressive systemic sclerosis, and Sjögren’s syndrome). Ankylosing spondylitis was not included in either group because the items in the original EIA detection tool were not designed specifically for its detection. Systemic lupus erythematosus was also not included because the arthritis typically seen in it is very different from that observed in rheumatoid arthritis. The independent and significant variables associated with inflammatory arthritis versus non-inflammatory arthritis were evaluated with multivariate logistic regression analysis. The area under the receiver operating characteristic curve was used to evaluate the discriminative performance of the model. The Youden index J (sensitivity + specificity −1) was used to determine an optimal cut-off value. In addition, 95 % confidence intervals for sensitivity, specificity, positive predictive values, and negative predictive values were determined by the Wilson score interval.

A two-tailed *p* value of <0.05 was considered statistically significant. All statistical analyses were conducted using IBM SPSS Statistics software package, version 21.0 (IBM Corp., Armonk, NY, USA).

## Results

A total of 131 participants were enrolled in the study. The overall mean age was 51.6 years and 38.2 % of them were males. The age and sex distribution among the six groups are shown in Table 1. The mean ages of osteoarthritis (65.4 years) and rheumatoid arthritis (57.3 years) were significantly older than that of the controls (42.4 years). There were significantly more male participants in the ankylosing spondylitis group compared with the rheumatoid arthritis group, the osteoarthritis group, and the systemic autoimmune diseases group (progressive systemic sclerosis, Sjögren’s syndrome, and systemic lupus erythematosus).

**Table 1:** Demographic characteristic of participants (*N* = 131)

Variable	Total	Group						*p*
		Control	Rheumatoid arthritis	Osteoarthritis	SAD	Psoriatic arthritis	Ankylosing spondylitis	
	131 (100)	20 (15.3)	31 (23.7)	20 (15.3)	20 (15.3)	20 (15.3)	20 (15.3)	
Age (years)								<0.001
Mean (SD)	51.6 (15.1)	42.4 (14.5)^a^	57.3 (14.9)^b^	65.4 (10.2)^b^	47.2 (12.4)^abc^	49.5 (11.9)^abc^	44.6 (13.7)^abc^	
Sex								<0.001
Male	50 (38.2)	7 (35)^abc^	7 (23)^ab^	5 (25)^ab^	3 (15)^ab^	12 (60)^abc^	16 (80)^c^	
Female	81 (61.8)	13(65)	24 (77)	15 (75)	17 (85)	8 (40)	4 (20)	

Age across groups were compared using non-parametric Kruskal-Wallis test and Mann–Whitney test with Dunn-Sidak adjustment was applied to control for the probability of Type I errors in pairwise comparisons

Sex distribution across groups were compared using Fisher’s exact test and Dunn-Sidak adjustment was applied in the pairwise comparisons

*SAD* systemic autoimmune diseases including progressive systemic sclerosis, Sjögren’s syndrome, and systemic lupus erythematosus

Figures with different superscript were significantly different at *p* < 0.05

The 11 items of the Chinese EIA detection tool showed an acceptable internal consistency (KR-20 coefficient 0.78), indicating that this tool is a reasonably reliable instrument in producing consistent scores. As shown in Table 2, the κ statistic for assessing test-retest reliability ranged from 0.43 to 0.94.

**Table 2:** Test-retest reliability (κ statistic) of the 11 items in the Chinese version of the Early Inflammatory Arthritis detection tool (*N* = 74)

Item	Test-retest reliability
	κ statistic (95 % CI)
1. Do you have pain in your joints?	0.73 (0.57–0.89)
2. Do you have pain in your wrists and hands?	0.67 (0.50–0.84)
3. Are your hands or wrists swollen?	0.42 (0.24–0.60)
4. Do you have trouble making a fist?	0.53 (0.27–0.79)
5. Are your joints stiff in the morning?	0.76 (0.61–0.91)
6. From the time you wake in the morning, does it take more than 60 min for your joints to move more freely?	0.47 (0.16–0.78)
7. Are the same joints involved on both sides of your body?	0.76 (0.60–0.92)
8. Have important activities in your life been affected because of bone or joint problems, such as having difficulty with personal care or having to make a change regarding leisure or work activities?	0.55 (0.35–0.76)
9. Have you ever been told that you have rheumatoid arthritis?	0.81 (0.67–0.94)
10. Does anyone in your family have rheumatoid arthritis?	0.94 (0.82–1.00)
11. Have you been diagnosed with a rash called psoriasis?	0.86 (0.72–1.00)

Table 3 shows the Chinese EIA detection tool scores and the frequencies of presence of each item across the six groups. The median value of the score was the lowest, as expected, in the control group and was the highest in the rheumatoid arthritis group. The median value of the Chinese EIA detection tool score for rheumatoid arthritis was significantly higher than that of the control, systemic autoimmune diseases group (progressive systemic sclerosis, Sjögren’s syndrome, and systemic lupus erythematosus), and ankylosing spondylitis. The median value of the Chinese EIA detection tool score for osteoarthritis was not significantly different from the other groups except that of the controls. The frequencies of presence of the 11 items were significantly different across the groups for all of them except in item 6 and item 10.

**Table 3:** Frequency of presence of conditions in the Chinese version of the Early Inflammatory Arthritis detection tool (*N* = 131)

Score or item	Group						*p*
	Frequency (%)						
	Control	Rheumatoid arthritis	Osteoarthritis	SAD	Psoriatic arthritis	Ankylosing spondylitis	
	20 (15)	31 (24)	20 (15)	20 (15)	20 (15)	20 (15)	
Chinese EIA detection tool score	3.2 (6.1)	47.8 (22.4)	30.0 (14.8)	22.7 (25.5)	44.1 (22.7)	27.3 (16.7)	<0.001
Mean (S. D.) [median, minimum, maximum]	[0, 0, 18]^a^	[46, 9, 91]^b^	[27, 9, 55]^bd^	[9, 0, 91]^cd^	[46, 9, 82]^bc^	[27, 0, 64]^cd^	
1. Do you have pain in your joints?	2 (10)^a^	24 (78)^bc^	19 (95)^c^	8 (40)^ab^	13 (65)^bc^	12 (60)^bc^	<0.001
2. Do you have pain in your wrists and hands?	1 (5)^a^	20 (65)^bc^	6 (30)^ac^	8 (40)^ac^	13 (65)^bc^	4 (20)^ac^	<0.001
3. Are your hands or wrists swollen?	0 (0)^a^	17 (55)^b^	2 (10)^ac^	5 (25)^abc^	9 (45)^bc^	1 (5)^ac^	<0.001
4. Do you have trouble making a fist?	0 (0)	11 (36)	2 (10)	3 (15)	4 (20)	2 (10)	0.020
5. Are your joints stiff in the morning?	1 (5)^a^	18 (58)^b^	12 (60)^b^	10 (50)^b^	13 (65)^b^	15 (75)^b^	<0.001
6. From the time you wake in the morning, does it take more than 60 min for your joints to move more freely?	0 (0)	5 (16)	1 (5)	1 (5)	5 (25)	2 (10)	0.123
7. Are the same joints involved on both sides of your body?	0 (0)^a^	14 (45)^bc^	9 (45)^bc^	5 (25)^ac^	7 (35)^ac^	8 (40)^bc^	0.003
8. Have important activities in your life been affected because of bone or joint problems, such as having difficulty with personal care or having to make a change regarding leisure or work activities?	2 (10)^a^	17 (55)^bc^	9 (45)^ac^	1 (5)^a^	8 (40)^ac^	7 (35)^ac^	0.001
9. Have you ever been told that you have rheumatoid arthritis?	0 (0)^a^	29 (94)^b^	3 (15)^a^	4 (20)^a^	3 (15)^a^	4 (20)^a^	<0.001
10. Does anyone in your family have rheumatoid arthritis?	0 (0)	5 (16)	3 (15)	4 (20)	3 (15)	3 (15)	0.421
11. Have you been diagnosed with a rash called psoriasis?	1 (5)^a^	3 (10)^a^	0 (0)^a^	1 (5)^a^	19 (95)^b^	2 (10)^a^	<0.001

Chinese EIA detection tool score = sum of yes response in item 1 to 11, standardized to a range of 0 to 100

The Chinese EIA detection tool scores across groups were compared with non-parametric Kruskal-Wallis test and Mann–Whitney test with Dunn-Sidak correction was applied to control for the probability of Type I errors in pairwise comparisons

*SAD* systemic autoimmune diseases including progressive systemic sclerosis, Sjögren’s syndrome, and systemic lupus erythematosus

Figures with different superscript were significantly different at *p* < 0.05, adjusting for multiple comparisons with Dunn-Sidak correction

Table 4 shows the significant and independent factors associated with osteoarthritis, each of the four groups of rheumatic diseases, and control. Age was a significant and independent factor associated with control, rheumatoid arthritis, osteoarthritis, and ankylosing spondylitis. Male sex was associated with ankylosing spondylitis. Item 1 of the Chinese EIA detection tool (joint pain) was associated directly with osteoarthritis but inversely with the control group. Item 2 (hand/wrist pain) was associated directly with psoriatic arthritis but inversely with ankylosing spondylitis. Item 3 (hand/wrist swelling) was associated directly with rheumatoid arthritis but inversely with osteoarthritis. Item 5 (morning stiffness) was associated directly with ankylosing spondylitis but inversely with the control group. Item 6 (morning stiffness duration >60 min) was associated directly only with psoriatic arthritis. Item 8 (functional difficulty) was inversely associated with systemic autoimmune diseases. Item 9 (ever been told to have rheumatoid arthritis) was associated directly with rheumatoid arthritis but inversely with psoriatic arthritis. Finally, item 11 (psoriasis diagnosis) was associated inversely with the control group.

**Table 4:** Multivariate logistic regression analyses of control, rheumatoid arthritis, osteoarthritis, systemic autoimmune diseases, psoriatic arthritis, and ankylosing spondylitis (*N* = 131)

Variable	Group					
	odds ratio (95 % confidence interval)					
	Control	Rheumatoid arthritis	Osteoarthritis	SAD	Psoriatic arthritis	Ankylosing spondylitis
Age (per 10 years)	0.56 (0.35–0.92)	1.62 (1.03–2.55)	2.46 (1.46–4.14)	–	–	0.67 (0.45–0.98)
Sex (females as reference group)	–	–	–	–	–	7.43 (2.16–25.66)
1. Do you have pain in your joints?	0.11 (0.02–0.58)	–	19.47 (2.33–163.06)	–	–	–
2. Do you have pain in your wrists and hands?	–	–	–	–	8.34 (2.56–27.23)	0.17 (0.04–0.66)
3. Are your hands or wrists swollen?	–	5.26 (1.34–20.7)	0.10 (0.02–0.55)	–	–	–
4. Do you have trouble making a fist?	–	–	–	–	–	–
5. Are your joints stiff in the morning?	0.04 (0.01–0.38)	–	–	–	–	5.19 (1.41–19.04)
6. From the time you wake in the morning, does it take more than 60 min for your joints to move more freely?	–	–	–	–	11.05 (2.18–56.08)	–
7. Are the same joints involved on both sides of your body?	–	–	–	–	–	–
8. Have important activities in your life been affected because of bone or joint problems, such as having difficulty with personal care or having to make a change regarding leisure or work activities?	–	–	–	0.08 (0.01–0.64)	–	–
9. Have you ever been told that you have rheumatoid arthritis?	–	93.45 (17.73–492.53)	–	–	0.05 (0.01–0.32)	–
10. Does anyone in your family have rheumatoid arthritis?	–	–	–	–	–	–
11. Have you been diagnosed with a rash called psoriasis?	0.10 (0.01–0.98)	–	–	–	–	–
Nagelkerke R^2^	0.54	0.69	0.47	0.14	0.30	0.38

*SAD* systemic autoimmune diseases including progressive systemic sclerosis, Sjögren’s syndrome, and systemic lupus erythematosus

Table 5 shows the results of the multivariate logistic regression model of inflammatory arthritis versus non-inflammatory arthritis musculoskeletal disorders. Four items emerged as significant and independent factors and they included sex (adjusted OR = 6.94, *p* = 0.008), item 1 (joint pain) (adjusted OR = 0.23, *p* = 0.046), item 3 (hand/wrist swelling) (adjusted OR = 8.88, *p* = 0.004), and item 9 (ever been told to have rheumatoid arthritis) (adjusted OR = 16.38, *p* < 0.001). Using the Youden index-optimized cut-off point of 0.60 to rescale the threshold to 0, the logistic regression equation results in the following formula:

$$ \mathrm{inflammatory}\ \mathrm{arthritis}=\hbox{--} 1.28+\left(1.94\times \mathrm{sex}\right)\hbox{--} \left(1.45\times \mathrm{item}\ 1\right)+\left(2.18\times \mathrm{item}\ 3\right)+\left(2.80\times \mathrm{item}\ 9\right) $$

**Table 5:** A prediction model of inflammatory arthritis versus non-inflammatory arthritis musculoskeletal disorders (*N* = 80)

Variable in the prediction model of inflammatory arthritis versus non-inflammatory arthritis	odds ratio (95 % CI)	*p* value
Sex (female as reference group)	6.94 (1.66–29.08)	0.008
1. Do you have pain in your joints?	0.23 (0.06–0.98)	0.046
3. Are your hands or wrists swollen?	8.88 (2.04–38.59)	0.004
9. Have you ever been told that you have rheumatoid arthritis?	16.38 (3.93–68.30)	<0.001
Intercept of the model (β coefficient)	−0.64	–
Nagelkerke R^2^	0.529	–
Performance parameter of the prediction model		
Area under the ROC curve (95 % CI)	0.88 (0.80–0.96)	
Optimal cut-off point	0.60	
Youden index J	0.71	
Sensitivity (95 % CI)	0.84 (0.71–0.93)	
Specificity (95 % CI)	0.86 (0.68–0.96)	
Positive predictive value (95 % CI)	0.92 (0.79–0.97)	
Negative predictive value (95 % CI)	0.76 (0.57–0.88)	

The inflammatory arthritis group consisted of rheumatoid arthritis and psoriatic arthritis. The non-inflammatory arthritis musculoskeletal disorders group consisted of osteoarthritis, progressive systemic sclerosis, and Sjögren’s syndrome

Age, sex, and all 11 items of the Chinese EIA detection tool were evaluated in the multivariate logistic regression analysis

95 % CI: 95 % confidence interval; ROC: receiver operating characteristic

In this formula, the item variable takes on the value “1” if the response is yes and the value “0” if the response is no. For the variable sex, it takes on the value “1” for males and “0” for females. With the rescaling of the logistic regression equation, a positive result predicts an inflammatory arthritis case while a negative result predicts a non-inflammatory arthritis case. Regarding the prediction performance of the model, based on the Youden index-optimized cut-off value of 0.60, the model had a sensitivity of 0.84 and a specificity of 0.86 with a positive predictive value of 0.92 and a negative predictive value of 0.76.

## Discussion

The main aim of this study was to translate the English version of the EIA detection tool to Chinese and examine its clinimetric properties. Overall, the Chinese EIA detection tool showed an acceptable internal consistency and a reasonably satisfactory test-retest reliability. The EIA detection tool was previously translated and adapted for screening of early inflammatory arthritis in Arabic-speaking populations. In a study of 30 patients, the internal consistency showed an acceptable value of KR-20 of 0.87. The Spearman’s rank correlations between the Arabic EIA detection tool score was 0.90 with pain score and 0.80 with Disease Activity Score 28 [13]. We noted that the κ statistic value over the Chinese EIA detection tool item 3 (hand/wrist swelling), item 4 (trouble making a fist), item 6 (morning stiffness >60 min), and item 8 (functional difficulty) were all less than 0.6. Due to the majority of population were out-patients with musculoskeletal disorders, these inconsistencies may reflect the improvement after receiving medical treatment. In addition, the test-retest period, ranged from 14 to 49 days, used in this study is longer than the typical 2-week interval in test-retest studies. This may account for the lower reliability in some of the items in the Chinese EIA detection tool compared with the original tool [12].

In addition to focus on the clinimetric properties of the Chinese EIA detection tool on patients with rheumatoid arthritis, the present study also included patients with established diagnosis of osteoarthritis, ankylosing spondylitis, and systemic autoimmune diseases, which are common causes of joint pain often encountered in rheumatology clinics. Several points worth mentioning regarding the results of the multivariate logistic regression analyses of the independent Chinese EIA detection tool items associated patients with the musculoskeletal disorders (Table 4). As expected, item 9 (ever been told to have rheumatoid arthritis) was strongly associated with the rheumatoid arthritis group. In addition, hand/wrist swelling was significantly associated with the rheumatoid arthritis group but inversely associated with the osteoarthritis group. This item could be useful clinically for differentiating these two diseases.

Furthermore, we noted that Chinese EIA detection tool item 4, item 7, and item 10 were not associated with any clinical conditions. In item 4 (trouble make a fist), this item was reported to be a useful indicator in one study [11] but failed to do so in another study [12]. Regarding the symmetry of the affected joints (item 7), it should be noted that although symmetry is one of the clinical presentation according to the 1987 American College of Rheumatology classification criteria for rheumatoid arthritis [14], it was dropped from the 2010 American College of Rheumatology (ACR) and the European League Against Rheumatism (EULAR) Rheumatoid Arthritis Classification Criteria [15]. Findings from our study support this change. For item 10, although a family history of rheumatoid arthritis is known to be a strong risk factor for developing rheumatoid arthritis [16], it did not emerge as a significant factor in any of our prediction models. In our sample, 16 % of the patients with rheumatoid arthritis had a family history of rheumatoid arthritis, but 15–20 % of the patients with musculoskeletal disorders other than rheumatoid arthritis also had a family history of rheumatoid arthritis. In a study on the measurement properties of the EIA detection tool, the proportions of participants with a family history of rheumatoid arthritis ranged from approximately 16–34 % and were not significantly different among participants with early inflammatory arthritis, established inflammatory arthritis, non-inflammatory arthritis musculoskeletal conditions, and non-musculoskeletal conditions [12]. In addition, a register-based Swedish study showed that less than 3 % of the patients with rheumatoid arthritis had a first-degree relative diagnosed with rheumatoid arthritis [17]. Therefore, the relatively high proportion of patients with rheumatoid arthritis reported to have family member with rheumatoid arthritis is likely to be an overestimation as a result of family information bias [18].

The Chinese EIA detection tool could be useful for patients with ankylosing spondylitis and systemic autoimmune diseases (progressive systemic sclerosis, Sjögren’s syndrome, and systemic lupus erythematosus) because these diseases are often associated with joint pain and they need to be differentiated from inflammatory arthritis. As shown in Table 3, although both patients with inflammatory arthritis and systemic autoimmune diseases have similar elevated proportion of joint pain (accessed with item 1 and item 2) and morning stiffness (accessed with item 5), patients with systemic autoimmune diseases did not have increased functional difficulty (accessed with item 8). These findings are consistent with clinical observation that arthralgia is the main complaint of patients with systemic autoimmune diseases. Regarding ankylosing spondylitis, the Chinese EIA detection tool item 5 (Are you joints stiff in the morning?) was significantly associated with ankylosing spondylitis (Table 4). This result is consistent with the typical symptom of spine stiffness in the morning among patients ankylosing spondylitis. Although not specifically designed for, the Chinese EIA detection tool was able to differentiate patients with ankylosing spondylitis from the other conditions. It is likely that patients with ankylosing spondylitis interpreted the words “joints stiff” in item 5 as spine stiffness. Overall, the Chinese EIA detection tool appeared to be able to discriminate osteoarthritis, rheumatoid arthritis, psoriatic arthritis, and ankylosing spondylitis in our clinical sample.

Furthermore, a prediction model based on multivariate logistic regression analysis was developed with the diseases classified as either inflammatory arthritis or non-inflammatory arthritis (Table 5). The final model with four variables had an area under the receiver-operating characteristic curve of 0.88, indicating the model could accurately predict inflammatory arthritis. Our regression models were different from that reported by Tavares and colleagues [11], which could be explained by the differences in the study sample. We compared the patients with established diagnosis of inflammatory arthritis and non-inflammatory arthritis musculoskeletal disorders, with the latter group containing patients with osteoarthritis, progressive systemic sclerosis, and Sjögren’s syndrome. On the other hand, Tavares and colleagues [11] compared patients with inflammatory arthritis and non-inflammatory arthritis who were referred to rheumatologic care. Therefore, depending on the characteristics of the clinical setting, different prediction models may have to be developed accordingly.

Our study presents some limitations that deserve comment. First, all our patients already had established diagnosis of various musculoskeletal disorders for several years. Therefore, their responses to the items of the Chinese EIA detection tool might be affected by the duration of their diseases. Nevertheless, our results provided support for the usefulness of the tool in differentiating inflammatory arthritis versus non-inflammatory arthritis musculoskeletal conditions. Continued research is needed to establish the clinical utility of the Chinese EIA detection tool in patients at the early stage of their disease. The original English EIA detection tool is recommended for use among patients with at least 6 weeks but less than 52 weeks of musculoskeletal symptoms. Second, a culture difference exists as some patients were confused between the diagnosis of psoriasis and dermatophytosis. Third, the disease activity of our patients could be affected by their medication treatment. Nevertheless, our patients still exhibited clinical signs that are typical of their respective diseases.

## Conclusions

This study showed that the Chinese EIA detection tool has an acceptable internal consistency and is reasonably reliable. The full Chinese EIA detection tool appeared to be able to differentiate osteoarthritis, rheumatoid arthritis, psoriatic arthritis, and ankylosing spondylitis among each other. In addition, a multivariate logistic regression model based on three items of the Chinese EIA detection tool and sex was developed for differentiating inflammatory arthritis versus non-inflammatory arthritis musculoskeletal conditions with good prediction performance.

## Abbreviations

DMARD: Disease modifying antirheumatic drug
EIA: Early inflammatory arthritis
